# Experimental design and statistical rigor in phylogenomics of horizontal and endosymbiotic gene transfer

**DOI:** 10.1186/1471-2148-11-259

**Published:** 2011-09-16

**Authors:** John W Stiller

**Affiliations:** 1 Department of Biology, East Carolina University, Greenville, NC 27858, USA

## Abstract

A growing number of phylogenomic investigations from diverse eukaryotes are examining conflicts among gene trees as evidence of horizontal gene transfer. If multiple foreign genes from the same eukaryotic lineage are found in a given genome, it is increasingly interpreted as concerted gene transfers during a cryptic endosymbiosis in the organism's evolutionary past, also known as "endosymbiotic gene transfer" or EGT. A number of provocative hypotheses of lost or serially replaced endosymbionts have been advanced; to date, however, these inferences largely have been post-hoc interpretations of genomic-wide conflicts among gene trees. With data sets as large and complex as eukaryotic genome sequences, it is critical to examine alternative explanations for intra-genome phylogenetic conflicts, particularly how much conflicting signal is expected from directional biases and statistical noise. The availability of genome-level data both permits and necessitates phylogenomics that test explicit, *a priori *predictions of horizontal gene transfer, using rigorous statistical methods and clearly defined experimental controls.

## 

Although specific details of how plastids originated and spread among eukaryotes remain under debate [1-8], there is little doubt that extant photosynthetic taxa evolved through a very complicated process. There have been several independent primary origins of plastids from cyanobacterial endosymbionts [9,10], as well as undetermined numbers of secondary, tertiary, and perhaps higher order endosymbioses involving eukaryotic to eukaryotic plastid transfer (see [11-13] for reviews). If hypotheses that minimize the number of endosymbioses prove correct, plastids also have been lost on numerous occasions [1,14,15]. Finally, there have been a number of proposed and documented cases of serial replacement of endosymbionts from very different taxonomic sources [16-21].

Regardless of which of these scenarios turn out to be validated, it is clear that plastid endosymbioses have remodeled host cell genomes substantially through the process of "endosymbiotic gene transfer (EGT). EGT is a special case of horizontal gene transfer (HGT) involving concerted movement of many endosymbiont genes into the host cell nucleus [22]; its impacts have been dramatic (Figure 1A). For example, nearly 20% of genes in the *Arabidopsis *nuclear genome are derived from the cyanobacterial ancestor of chloroplasts [23]. Moreover, only about half of these genes appear to be related to chloroplast function; the rest either replaced original eukaryotic homologs or were adapted to entirely novel functions in the host cell's metabolism. Overall contributions from EGT vary among photosynthetic taxa [24] and eukaryotic to eukaryotic EGT is more difficult to quantify [22], partly because phylogenetic relationships among major groups remain unclear. Nevertheless, there is no doubt that large scale EGT has been a feature of both primary and higher order plastid endosymbioses [22]. Transferred genes that are not related to plastid function are of particular interest because many should remain under strong purifying selection, even if photosynthesis and the plastid itself are lost. Effectively, they offer the promise of finding "footprints" [25] of lost plastids or long-term, stable endosymbionts when no cytological or metabolic evidence remains (Figure 1B).

It has become popular to look for EGT "footprints" in genomes of heterotrophic eukaryotes, particularly those from which current evolutionary models suggest plastids could have been lost. For example, "algal" genes in both ciliates and oomycetes have been cited as support for the "chromalveolate" model of plastid evolution [1,16,26,27]; that is, a single secondary origin of plastids in the ancient ancestor of a large and diverse assembly or organisms, with subsequent plastid losses from extant heterotrophic taxa. Other phylogenomic investigations of HGT have offered provocative new evolutionary hypotheses about the reticulate history of eukaryotic photosynthesis. They also raise novel problems, both methodological and computational, that require new analytical approaches; to date, these problems have not been addressed in most phylogenomic investigations of HGT/EGT.

## The complexity of phylogenetic conflict within genomes

Searching for possible cases of HGT, or concerted HGT that could reflect EGT, usually involves an automated computational pipeline to uncover conflicting phylogenetic signals across the genome [28,29]. Genes that cluster strongly with one or another algal taxon are interpreted as potential examples of EGT; that is, genes transferred to the nucleus over a long association between endosymbiont and host. Because of uncertainties about current models of plastid evolution, and the unreliability of individual gene trees (see below), genes from different algal taxa often are counted collectively as evidence of EGT from a lost plastid [19,26,27].

These kinds of phylogeny-based approaches implicitly assume that strong support for clustering any given gene with an algal clade is reliable evidence of gene transfer from a member of that algal group. When mining data sets as large and complex as a typical eukaryotic genome, however, it is essential to consider alternative phylogenetic models, as well as known sources of intra-genomic phylogenetic conflict that can result in aberrant but strongly supported trees [30-32]. A number of potential problems, both biological and statistical, should be addressed explicitly and rigorously in genome-wide analyses of HGT, particularly before hypothesizing new and complicated scenarios like lost plastid endosymbiosis.

### 1. Current evolutionary models require further scrutiny

Assumptions of HGT generally are made for genes that produce phylogenies different from expected relationships of the organisms in question. Expected relationships, in turn, are based on "generally accepted" models recovered in previous phylogenetic and phylogenomic studies. Beyond the potential for circular reinforcement of prior results, there are two major problems with this approach. First, most popular scenarios of eukaryotic evolution were developed without consideration of the enormous impact of endosymbioses on algal and plant genomes. Consequently, some tree-building signals they are based upon could result from concerted gene transfers rather than evolutionary relatedness. For example, recovery of the Archaeplastida or Plantae, comprising green algae and plants, red algae and glaucophytes in many phylogenomic studies [33-35] could reflect relationships through endosymbiosis rather than direct descent [3,36]. Likewise, a strong affinity between Viridiplantae and Stramenopiles recovered through phylogenomic pipelines [16,34,37] could indicate a closer evolutionary relationship than hitherto recognized, and not EGT as has been assumed. At this relatively early stage of eukaryote-wide phylogenomics, strongly conflicting tree-building signals bear consideration as results that could falsify current phylogenetic models; they should not be interpreted through an uncritical lens of one or another preferred scenario.

The second problem with over-reliance on working models of eukaryotic evolution is that many of them are poorly supported. Overall resolution of relationships among major eukaryotic groups remains poor, and there is a lack of congruity among studies depending on data sets and methodologies employed. It can require the cumulative signal from hundreds of genes and tens thousands of aligned positions to recover strong support for nodes that are fundamental to understanding relationships among major photosynthetic lineages [33,35,38]. For some key relationships, there is no current consensus. As mentioned above, a number of presumptions of EGT have been based on the "chromalveolate" model of evolution of the red plastid lineage [14]. Yet "chromalveolates" never have enjoyed clear phylogenetic support [4,7,39], and some of the largest and best-resolved phylogenomic studies to date [38,40,41], as well as the few studies designed explicitly to test it [42,43], reject this model of plastid evolution. Even when key relationships are strongly supported, major questions remain as to whether the tree-building signal reflects history or the cumulative effects of biases within and among genomes [3,44,45]. Current models of eukaryotic relationships are working hypotheses and subjects of ongoing controversy, and require further rigorous testing; it is premature to layer major new scenarios of EGT and lost endosymbioses on top of them.

### 2. The need for objective bases for invoking EGT

Even assuming a given popular model of evolution is historically accurate, large numbers of algal genes could have arrived via more pedestrian processes of HGT; for example, because algae were a common prey item over long periods of the organism's evolution. Any phagotrophic ancestor that could have adopted an algal endosymbiont, must also have been eating algae on a regular basis. There is no obvious threshold for determining what level of phylogenetic signal implies repeated cases of HGT versus gene transfer from a lost endosymbiosis. Consequently, invoking EGT generally is a subjective decision based on preconceptions about the organism's past. When many algal genes were found in the choanoflagellete *Monosiga*, they were interpreted as likely products of HGT from prey organisms, because no popular model of evolution assumes plastids were lost from ancestral opisthokonts [46]. When comparable or fewer numbers of algal genes were found in ciliates and oomycetes, they were interpreted as evidence of a lost endosymbiosis under an assumption of the "chromalveolate" model of plastid evolution [26,27]. If phylogenomic investigations are to be rigorous, scientific inquiries into EGT and plastid loss, there is a clear need to establish more objective criteria for determining whether a foreign gene complement is greater than expected from more typical kinds of HGT.

### 3. All strong phylogenetic conflicts are not evidence of HGT

**Figure 1:**
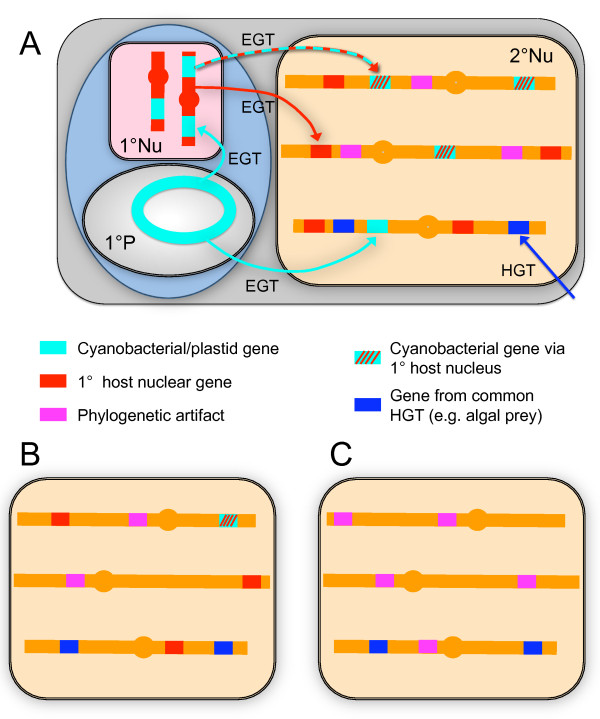
**Endosymbiotic gene transfer and other potential explanations for finding "algal" genes in any given genome**. **A**. Depicts the complexity of genome remodeling in typical secondary endosymbioses, particularly the massive transfer of genes from the primary plastid alga to its new host cell nucleus. Most genes from the original primary (cyanobacterial) plastid (1P) endosymbiont already made their way into the primary host nucleus (1Nu) via EGT. These genes are transferred, in turn, to the secondary host's nucleus (2Nu), if they are essential for plastid function. Some genes still present in the primary plastid genome could be transferred directly to the secondary host nucleus. Genes from the primary alga's nucleus that are not related to plastid function can be transferred to the secondary host, either replacing original homologs or adding novel functions to the host's metabolism. In addition, for as long as the secondary host was or remains phagotrophic, many additional "algal" genes could accumulate by more typical HGT from prey items. Finally, an unknown fraction of genes in the secondary host's genome are recovered with algal clades because of phylogenetic, tree-building artefacts. For more extensive reviews of eukaryotic EGT/HGT, see references [25,63]. **B**. Expectation if the secondary plastid is lost during the subsequent evolution of the algal genome shown in panel A. "Algal" genes directly related to plastid function are likely to accumulate null mutations and be lost, but many that were adapted to functions unrelated to photosynthesis should remain under strong purifying selection and be retained in the genome. These genes could represent a "footprint" of the past endosymbiosis, if they provide a significantly stronger phylogenetic signal than is expected from other known sources of tree-building conflicts, such as common HGT or phylogenetic artefacts. **C**. Panel shows a nucleus containing a comparable number of genes that cluster with algal sequences in phylogenetic analyses, but in this case most represent phylogenetic artefacts and none are from EGT. It is critical that investigations of EGT be designed to test explicitly among alternative, plausible explanations for the presence "algal" genes.

The thorniest problem with *a posteriori *interpretations of conflicting gene trees is that differences in evolutionary history are not the only, and perhaps not the predominant source of phylogenetic conflict within any given genome (Figure 1). Conflicts among individual gene trees are caused by a variety of stochastic, directional and as yet unidentified processes [47]; collectively these lead to phylogenetic artefacts such as the well-characterized problem of "long-branch attraction" [48]. Differences in evolutionary rates and biases in nucleotide, codon and amino acid compositions have long been recognized as common sources of phylogenetic artefacts [31]. Similarities in lifestyle (e.g. autotrophic versus heterotrophic) also can select for genome-level convergence, both in the varieties and sizes of gene families present, as well as at the levels of nucleotide or amino acid composition [49,50].

Years of research have demonstrated that individual gene trees are unreliable, particularly at the great evolutionary distances considered in studies of EGT. The small subunit (SSU or 16S-like) of the ribosome is the poster child for the severity of this problem. Considered the "gold-standard" for two decades of phylogenetic investigations of the tree of life [51], and incorporated into basic biology textbooks in the late 1990s, SSU rDNA trees were found to contain many inaccurate nodes, often with strong statistical support [52-54]. This was not because SSU rDNA is a "bad" phylogenetic marker; in fact, it remains among the most useful individual genes for examining the global tree of life. Rather, it highlights a consistent problem with sequence-based trees at deep phylogenetic levels; they generally (if not always) contain at least some erroneously placed branches. This problem is magnified substantially when conflicting gene trees are considered across whole genome data sets [32].

Some causes of tree-building conflicts, such as compositional biases and rate variation among sites, generally are factored into model-based phylogenetic approaches. Problems of covarions or heterotachy (residues in sequences do not maintain the same relative rates across sequences and through time) have proven much more difficult to model accurately, much less to incorporate into tree-building algorithms [55]. These factors are implicated specifically as contributing to the difficulties in resolving relationships among photosynthetic organisms [45,56]. In fact, discrepancies between phylogenetic models of sequence evolution, versus the biochemical reality deduced from experimentally solved protein tertiary structures, suggest that few internal nodes should be considered reliable at the evolutionary distances considered in most studies of EGT [57].

Additional biases in phylogenetic inference can arise from sampling tendencies of past researchers. For example, it has been shown that total phylogenetic affinity of a genome to those of distant taxonomic groups can depend on the relative number of sequences available from different groups in targeted databases, and that this similarity can mimic patterns that emerge from known evolutionary relatedness [43]. Inherent conflicts among individual gene trees are further exacerbated at the scale of whole genomes by inaccurate or incomplete annotation of many sequences, as well as the potential for external contamination, when incompletely assembled genomes or EST databases are run through automated pipelines. While annotation-related factors probably dampen out of total phylogenetic signal across a genome, they could strongly impact an unknown fraction of individual gene trees.

## What is the significance of finding algal genes in any given genome?

These cumulative problems associated with large and complex genomic datasets beg the question, what is the significance of finding a few dozen or even a few hundred "algal" sequences in a typical eukaryotic genome of perhaps 15000 genes (Figure 1B-C)? Put another way, if only one in a thousand of those genes cluster artificially with an algal sequence, 15 "algal" genes would be inferred. At one in a hundred, that number would be 150, certainly more than enough to be considered strong evidence of EGT using current approaches. It could be argued that either of these numbers overestimates the potential for directional phylogenetic artefacts. On the other hand, it is equally valid to assume that both are underestimates. Either supposition is subjective given the current state of knowledge about both intra-genome phylogenetic conflict and broad-scale eukaryotic relationships. At present, there may be no objective way to distinguish between a strong phylogenetic artefact and *bona fide *genetic transfer for any number of individual genes identified by an automated HGT pipeline.

## A more rigorous approach to analyzing signal from HGT

The inherent uncertainties in gene trees, along with the sheer number of them associated with a typical eukaryotic genome, make it essential to develop approaches that distinguish among potential sources of phylogenetic conflict. To address this problem, genome-level investigations should apply rigorous, hypothesis-based research plans, and employ clear and explicit statistical tests with appropriate controls. As an initial contribution toward developing such approaches, four major criteria should be considered in future phylogenomic investigations of EGT/HGT:

### 1. A posteriori *interpretations of genome-level conflicts among gene trees should not be considered completed scientific studies*

**Figure 2:**
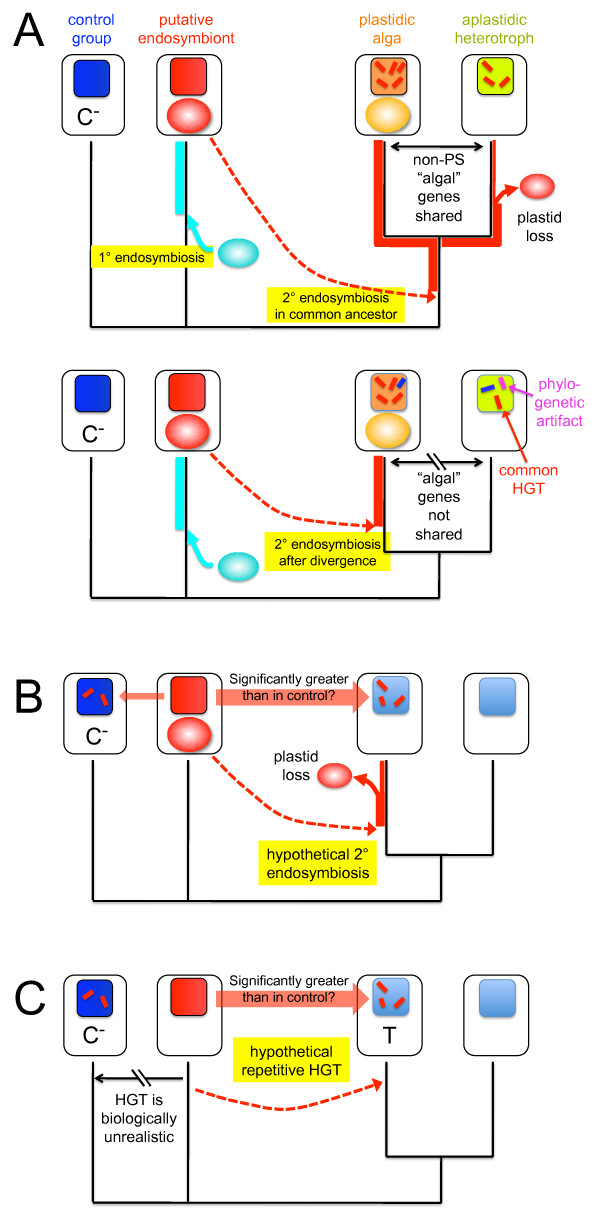
**Testing specific predictions of competing hypotheses to explain conflicting gene trees**. **A**. Patterned after an explicit test of the chromalveolate model of plastid evolution [43], the two hypothetical scenarios show mutually exclusive predictions about "algal" genes in a heterotrophic protist's genome based on two alternative evolutionary hypotheses. The upper scenario of a more ancient endosymbiosis predicts that genes from the secondary endosymbiont, those unrelated to plastid function, should be shared between the aplastidic heterotroph and its algal sister taxon. The lower scenario of a later, taxon-specific plastid origin, predicts that "algal" genes from the heterotroph are products of common HGT or phylogenetic artefacts and, therefore, should not be shared with the photosynthetic neighbor relative to a negative control (C^-^). The control is a taxon generally agreed to be unrelated, phylogenetically or through endosymbiosis, to either the host or endosymbiont lineages. If the shared phylogenetic signal from the putative endosymbiont is not significantly greater than from the control group, then there is no objective basis for advancing EGT as an explanation for apparent "algal" genes in the heterotroph's genome. **B**. EGT versus HGT in a heterotrophic taxon. In this case, a rigorous test could be whether there are significantly more "algal" genes in the organism of interest than in phagotrophic control taxa with no presumed history of EGT. If there is not a significantly greater signal of HGT from the presumed endosymbiont in the target genome than in the control taxa, then algal genes are consistent with HGT or phylogenetic artefacts and EGT is not supported. **C**. The same approach could be used to test whether repetitive HGT is a superior hypothesis to phylogenetic artefacts by examining control taxa that should have had little to no opportunity to take up DNA from the organism in question, based on their presumed ecological and evolutionary histories. If it is biologically unreasonable to expect common products of HGT in the control genome (C^-^), and there is comparable signal present as in the target genome (T), then HGT does not rise above the null hypothesis of signal from statistical biases and/or noise across genome-level data.

Clear *a priori *hypotheses should be in place before an analysis is undertaken. They should include specific predictions that distinguish among competing hypotheses (Figure 2A). For example, the chromalveolate model of plastid evolution predicts that "algal" genes from EGT (those unrelated to plastid function) should be shared between ochrophyte algae and their heterotrophic relatives like oomycetes and labyrinthulids. In contrast, an endosymbiosis only in ochrophytes predicts that phylogenetic signal from EGT should not be shared with these other groups. A recent statistical test of these two conflicting models rejected the hypothesis that "red algal" genes present in either diatom or oomycete genomes are shared between the two [43]. In cases where automated pipelines uncover strong new intra-genomic tree-building conflicts, these should be investigated using similar approaches that distinguish clearly between EGT and other reasonable explanations. New and complicated evolutionary models based only on gene tree conflicts should not be advanced without explicit experimental attempts to falsify them.

### 2. Phylogenetic signal from different algal taxa should not be grouped together as evidence of a lost plastid

Proposed signal from EGT should be limited to the specific taxon from which it is suspected to have originated. If enough uncertainty exists among phylogenies to require clustering algal genes to uncover evidence of EGT, then these phylogenies also are too uncertain to be the basis of a complex new evolutionary scenario. Moreover, whether algal genes from different sources are interpreted as evidence of one endosymbiosis [26,27] or multiple, serial endosymbioses [16,19], appears to be an entirely subjective decision at present.

### 3. Unambiguous statistical tests should be used to distinguish among sources of phylogenetic conflict in a genome

Whole genome data both require and permit new methodological approaches to deal with biases that lead to strong conflicts among individual gene trees [32]. A great deal of effort has gone into improving phylogenetic methods to take these biases into account on a gene by gene basis; however, very little work has focused on how to employ more classical statistical approaches that do not rely on modeling unknown or ambiguous parameters of sequence evolution across the entire genome [32].

Large genomes containing thousands of genes are tractable data sets for such tests. For example, relative numbers of individual phylogenies or significant alignment scores can be treated as data in Fisher exact or other statistical tests of specific predictions of competing hypotheses (Figure 2A-C) [43]. Probabilistic approaches that examine gene tree concordance across large, multi-gene data sets also have been used to examine HGT [58-60], and explicit statistical tests for EGT could be incorporated into these methods. Likewise, phylogenetic networks [61] rather than tree-by-tree interpretations can provide a statistical framework for examining intra-genomic tree-building conflicts as evidence of EGT. In addition to permitting direct tests of explicit evolutionary hypotheses, such statistical approaches also can account for size variation among genome data sets, both of the organism under investigation and of those targeted in automated pipelines [43].

### 4. Appropriate controls should be employed in all genome-level investigations of putative EGT

Because conflicts among gene trees are known to arise from a range of biological processes and statistical biases [30-32,62], it is essential to frame investigations of EGT in ways that provide rigorous comparisons of alternative hypotheses. An example of how to approach this problem would be to use genomes from heterotrophic/phagotrophic groups like choanoflagellates, that is, lineages believed never to have harbored a plastid, as a control for expected signal from common HGT (Figure 2B). Control organisms should be chosen that are unrelated to either the presumed endosymbiont or the host lineage. If significantly greater numbers of "algal" genes are present than in control genomes, then EGT is a viable hypothesis to consider over the alternative (and simpler) biological model.

As much as possible, control genomes should be chosen that are comparable in size, and likely to have a similar evolutionary history to the target genome, both biologically and ecologically. Because organisms vary in their rates of uptake of foreign genes, no single genome can provide a standard threshold, past which signal from concerted HGT is strong enough to indicate something more unusual like a lost plastid. With the coming availability of multiple genomes from diverse heterotrophic groups, however, it should be possible to define a mean and distribution of expected signal from more common forms of HGT, against which proposals of EGT can be compared statistically.

Although a somewhat more difficult problem to address, control genomes also should be employed, whenever possible, when mining whole genome data for cases of concerted HGT not presumed to be from EGT (Figure 2C). Organisms for which there is no biological basis to expect horizontal gene exchange can serve as controls for phylogenetic conflicts arising from tree-building artefacts. For example, if the genome from an exclusively and historically marine lineage contains genes from similarly exclusive terrestrial organisms, with no apparent vector between them, then these associations are much more likely to be phylogenetic artefacts than actual cases of HGT.

## Conclusions

The advent of comparative genomics across eukaryotic diversity necessitates a shift in methodologies applied to studies of gene transfer. With the amount and complexity of intra-genome phylogenetic signal emerging from investigations to date, it is critical to apply rigorous tests of explicit, mutually exclusive hypotheses to explain conflicts among gene trees. For a typical eukaryotic genome, trees can be found to support any number of different scenarios of concerted HGT, including multiple serial plastid replacements in many cases. If every strongly supported but aberrant gene tree is accepted, uncritically, as a case of gene transfer, inferences of HGT/EGT will become unfalsifiable hypotheses. Whether to accept or reject a given scenario as too complex, or biologically unrealistic, would be an entirely subjective exercise based on presumptions of individual researchers. Addressing these issues is particularly important with respect to EGT, because most current scenarios of plastid evolution assume that plastids are much easier to lose than to gain, an assumption that does not appear to be supported by empirical patterns of evolution in established photosynthetic lineages [4,39].

To prevent excessively complex, mutually contradictory, and potentially unfalsifiable scenarios from accumulating in the evolutionary literature, researchers, reviewers and editors should insist on rigorous hypothesis testing in genome-level investigations of HGT. The examples of statistical approaches and controls highlighted here may not be relevant to any given study. The important mutually exclusive predictions to test, how statistical tests should be designed, and what control groups are appropriate, will be specific to each individual investigation. It is critical, therefore, that researchers begin to pay as much attention to hypothesis-based experimental design and new statistical approaches to analyzing genome level data, as they have in developing methods to uncover genome-scale conflicts among gene trees.
